# 4-Hydrazinyl­idene-1-methyl-3*H*-2λ^6^,1-benzothia­zine-2,2-dione

**DOI:** 10.1107/S1600536811027577

**Published:** 2011-07-16

**Authors:** Muhammad Shafiq, Islam Ullah Khan, Muhammad Zia-ur-Rehman, Muhammad Nadeem Arshad, Abdullah M. Asiri

**Affiliations:** a Materials Chemistry Laboratory, Department of Chemistry, GC University, Lahore 54000, Pakistan; b Applied Chemistry Research Center, PCSIR Laboratories Complex, Ferozpur Road, Lahore 54600, Pakistan; c X-ray Diffraction and Physical Laboratory, Department of Physics, School of Physical Sciences, University of the Punjab, Quaid-e-Azam Campus, Lahore 54590, Pakistan; d The Center of Excellence for Advanced Materials Research, King Abdul Aziz University, Jeddah, PO Box 80203, Saudi Arabia

## Abstract

In the title compound, C_9_H_11_N_3_O_2_S, the thia­zine ring adopts a half-chair conformation. In the crystal structure N—H⋯N hydrogen bonds connect two mol­ecules into a centrosymmetric dimer, forming an *R*_2_^2^(6) ring motif. These dimers are further connected into chains by N—H⋯O and C—H⋯O hydrogen bonds.

## Related literature

For the synthesis of the title compound, see: Shafiq *et al.* (2011*b* ▶). For information on 1,2 and 2,1-benzothia­zine, see: Shafiq *et al.* (2011*a* ▶); Arshad *et al.* (2011 ▶). For related structures, see: Tahir *et al.* (2008 ▶); Khan *et al.* (2010 ▶); Shafiq *et al.* (2009 ▶); Arshad *et al.* (2009 ▶). For graph-set notation of hydrogen bonds, see: Bernstein *et al.* (1995 ▶).
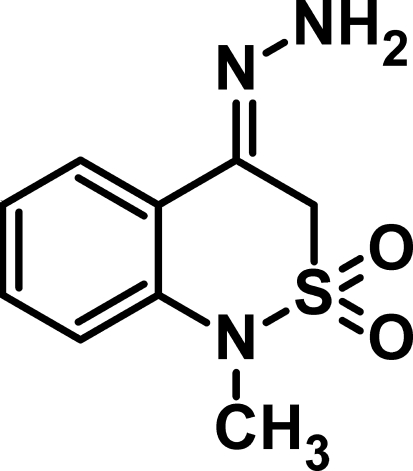


## Experimental

### 

#### Crystal data

C_9_H_11_N_3_O_2_S
*M* *_r_* = 225.27
Monoclinic, 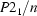

*a* = 6.6643 (2) Å
*b* = 9.6834 (3) Å
*c* = 15.5890 (5) Å
β = 97.699 (1)°
*V* = 996.94 (5) Å^3^
*Z* = 4
Mo *K*α radiation
μ = 0.31 mm^−1^
*T* = 296 K
0.41 × 0.22 × 0.18 mm

#### Data collection

Bruker Kappa APEXII CCD diffractometer
Absorption correction: multi-scan (*SADABS*; Bruker, 2007 ▶) *T*_min_ = 0.884, *T*_max_ = 0.947
8966 measured reflections
2426 independent reflections
2114 reflections with *I* > 2σ(*I*)
*R*_int_ = 0.020

#### Refinement

*R*[*F* ^2^ > 2σ(*F* ^2^)] = 0.036
*wR*(*F* ^2^) = 0.111
*S* = 0.93
2426 reflections
143 parameters
H atoms treated by a mixture of independent and constrained refinement
Δρ_max_ = 0.30 e Å^−3^
Δρ_min_ = −0.29 e Å^−3^

### 

Data collection: *APEX2* (Bruker, 2007 ▶); cell refinement: *SAINT* (Bruker, 2007 ▶); data reduction: *SAINT*; program(s) used to solve structure: *SHELXS97* (Sheldrick, 2008 ▶); program(s) used to refine structure: *SHELXL97* (Sheldrick, 2008 ▶); molecular graphics: *ORTEP-3 for Windows* (Farrugia, 1997 ▶) and *PLATON* (Spek, 2009 ▶); software used to prepare material for publication: *WinGX* (Farrugia, 1999 ▶) and *PLATON*.

## Supplementary Material

Crystal structure: contains datablock(s) I, global. DOI: 10.1107/S1600536811027577/bt5565sup1.cif

Structure factors: contains datablock(s) I. DOI: 10.1107/S1600536811027577/bt5565Isup2.hkl

Supplementary material file. DOI: 10.1107/S1600536811027577/bt5565Isup3.cml

Additional supplementary materials:  crystallographic information; 3D view; checkCIF report

## Figures and Tables

**Table 1:** Hydrogen-bond geometry (Å, °)

*D*—H⋯*A*	*D*—H	H⋯*A*	*D*⋯*A*	*D*—H⋯*A*
N3—H1*N*⋯O1^i^	0.86 (2)	2.46 (2)	3.221 (2)	147.7 (17)
N3—H2*N*⋯N2^ii^	0.790 (19)	2.376 (19)	3.094 (2)	151.8 (19)
C8—H8*A*⋯O1^i^	0.97	2.59	3.4178 (19)	144

Symmetry codes: (i) 
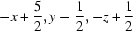
; (ii) 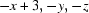.

**Fig. 1.**
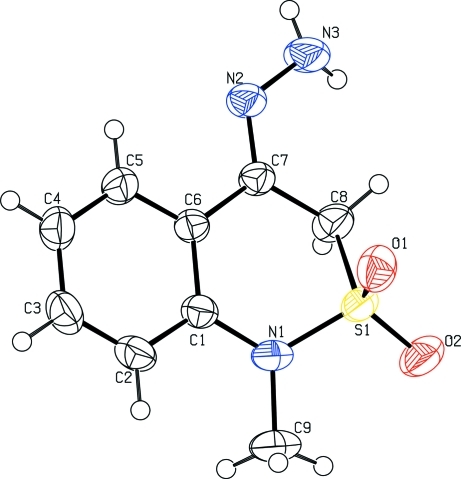
The molecular structure of the title compound with displacement ellipsoids drawn at 50% probability level.

**Fig. 2.**
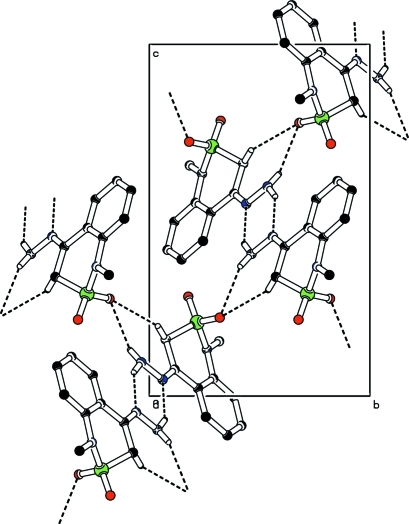
Perspective view showing the dimers and hydrogen bonding via dashed lines, hydrogen atoms not involved in hydrogen bonding have been omitted for clarity.

## supplementary crystallographic information

### Comment

We are already engaged in the synthesis (Shafiq *et al.*, 2011*a*), (Arshad *et al.*, 2011) and crystallographic studies of 1,2- & 2,1-benzothiazine molecules (Arshad *et al.*, 2009), (Shafiq *et al.*, 2009). Here in, we report the crystal structure of hydrazide (I), synthesized from 1-methyl-1*H*-2,1-benzothiazin-4(3*H*)-one 2,2-dioxide (II) (Tahir *et al.*, 2008).

In the crystal structure of title compound, the bond lengths and angles are almost similar to structurally similar molecules (II) and 1-propyl-1*H*-2,1-benzothiazin-4(3*H*)-one 2,2-dioxide (III) (Khan *et al.*, 2010). The fused aromatic and thiazine rings are twisted at dihedral angle of 11.13 (8)°. The thiazine ring (C1/C6/C7/C8/S1/N1) adopted the sofa shape with the r. m. s. deviavtion of fitted atoms of 0.2380Å showing the maximum deviation for the S1 (0.3969 (8)Å) &  C8 (0.2687 (7)Å). The molecules in the crystal structure dimerized through N—H···N hydrogen bonding forming six-membered *R*_2_^2^(6) ring motif (Bernstein *et al.*, 1995). There are C—H···O and N—H···N type interactions which connect the dimers in zig-zag mode along *c* axis and *a* axis and generate another seven membered ring motif *R*_2_^1^(7).

### Experimental

The synthesis of title compound have already been reportrd (Shafiq *et al.*, 2011*b*). Suitable crystals were grown in dry ethanol under slow evaporation.

### Refinement

H-atoms bonded to C were positioned with idealized geometry with C—H = 0.93 Å for aromatic, C—H = 0.96 Å for methyl group and C—H = 0.97 Å for methylene group and were refined using a riding model with *U*_iso_(H) = 1.2 *U*_eq_(C) for aromatic and methylene and with *U*_iso_(H) = 1.5 *U*_eq_(C) for methyl carbon atoms. The coordinates of the H atoms bonded to N were refined with *U*_iso_(H) = 1.2 *U*_eq_(N). Four reflections (-1 0 1, 0 1 1, 0 0 2 and -1 0 9) have been omitted in final refinement.

### Crystal data

**Table:**

C_9_H_11_N_3_O_2_S	*F*(000) = 472
*M**_r_* = 225.27	*D*_x_ = 1.501 Mg m^−^^3^
Monoclinic, *P*2_1_/*n*	Mo *K*α radiation, λ = 0.71073 Å
Hall symbol: -P 2yn	Cell parameters from 5698 reflections
*a* = 6.6643 (2) Å	θ = 3.7–28.3°
*b* = 9.6834 (3) Å	µ = 0.31 mm^−^^1^
*c* = 15.5890 (5) Å	*T* = 296 K
β = 97.699 (1)°	Needle, yellow
*V* = 996.94 (5) Å^3^	0.41 × 0.22 × 0.18 mm
*Z* = 4	

### Data collection

**Table:**

Bruker Kappa APEXII CCD diffractometer	2426 independent reflections
Radiation source: fine-focus sealed tube	2114 reflections with *I* > 2σ(*I*)
graphite	*R*_int_ = 0.020
φ and ω scans	θ_max_ = 28.3°, θ_min_ = 3.7°
Absorption correction: multi-scan (*SADABS*; Bruker, 2007)	*h* = −6→8
*T*_min_ = 0.884, *T*_max_ = 0.947	*k* = −11→12
8966 measured reflections	*l* = −19→20

### Refinement

**Table:**

Refinement on *F*^2^	Primary atom site location: structure-invariant direct methods
Least-squares matrix: full	Secondary atom site location: difference Fourier map
*R*[*F*^2^ > 2σ(*F*^2^)] = 0.036	Hydrogen site location: inferred from neighbouring sites
*wR*(*F*^2^) = 0.111	H atoms treated by a mixture of independent and constrained refinement
*S* = 0.93	*w* = 1/[σ^2^(*F*_o_^2^) + (0.0763*P*)^2^ + 0.3098*P*] where *P* = (*F*_o_^2^ + 2*F*_c_^2^)/3
2426 reflections	(Δ/σ)_max_ < 0.001
143 parameters	Δρ_max_ = 0.30 e Å^−^^3^
0 restraints	Δρ_min_ = −0.29 e Å^−^^3^

### Special details

**Table:**

Geometry. All s.u.'s (except the s.u. in the dihedral angle between two l.s. planes) are estimated using the full covariance matrix. The cell s.u.'s are taken into account individually in the estimation of s.u.'s in distances, angles and torsion angles; correlations between s.u.'s in cell parameters are only used when they are defined by crystal symmetry. An approximate (isotropic) treatment of cell s.u.'s is used for estimating s.u.'s involving l.s. planes.
Refinement. Refinement of F^2^ against ALL reflections. The weighted R-factor wR and goodness of fit S are based on F^2^, conventional R-factors R are based on F, with F set to zero for negative F^2^. The threshold expression of F^2^ > 2σ(F^2^) is used only for calculating R-factors(gt) etc. and is not relevant to the choice of reflections for refinement. R-factors based on F^2^ are statistically about twice as large as those based on F, and R- factors based on ALL data will be even larger.

### Fractional atomic coordinates and isotropic or equivalent isotropic displacement parameters (Å^2^)

**Table:**

	*x*	*y*	*z*	*U*_iso_*/*U*_eq_	
C1	0.8692 (2)	0.28978 (14)	0.04960 (9)	0.0354 (3)	
C2	0.7617 (2)	0.38627 (17)	−0.00433 (10)	0.0460 (4)	
H2	0.6510	0.4306	0.0138	0.055*	
C3	0.8167 (3)	0.41727 (17)	−0.08437 (11)	0.0491 (4)	
H3	0.7420	0.4811	−0.1200	0.059*	
C4	0.9815 (3)	0.35393 (17)	−0.11144 (10)	0.0468 (4)	
H4	1.0209	0.3765	−0.1647	0.056*	
C5	1.0884 (2)	0.25656 (16)	−0.05909 (10)	0.0409 (3)	
H5	1.1990	0.2134	−0.0781	0.049*	
C6	1.0349 (2)	0.22102 (13)	0.02180 (9)	0.0328 (3)	
C7	1.1508 (2)	0.11464 (13)	0.07523 (8)	0.0341 (3)	
C8	1.0878 (3)	0.07413 (16)	0.16103 (10)	0.0454 (4)	
H8A	1.2040	0.0386	0.1987	0.054*	
H8B	0.9866	0.0017	0.1524	0.054*	
C9	0.6206 (3)	0.3164 (2)	0.15568 (14)	0.0609 (5)	
H9A	0.6343	0.4141	0.1652	0.091*	
H9B	0.5906	0.2723	0.2076	0.091*	
H9C	0.5128	0.2991	0.1096	0.091*	
N1	0.80990 (19)	0.26108 (15)	0.13215 (9)	0.0446 (3)	
N2	1.30403 (19)	0.06040 (13)	0.04671 (8)	0.0427 (3)	
N3	1.4169 (2)	−0.03646 (17)	0.09606 (11)	0.0565 (4)	
O1	1.14407 (18)	0.31926 (13)	0.22310 (7)	0.0515 (3)	
O2	0.8941 (2)	0.17659 (15)	0.28274 (8)	0.0587 (3)	
S1	0.98824 (5)	0.21730 (4)	0.20972 (2)	0.03865 (15)	
H1N	1.357 (3)	−0.087 (2)	0.1305 (13)	0.046*	
H2N	1.494 (3)	−0.070 (2)	0.0678 (12)	0.046*	

### Atomic displacement parameters (Å^2^)

**Table:**

	*U*^11^	*U*^22^	*U*^33^	*U*^12^	*U*^13^	*U*^23^
C1	0.0314 (6)	0.0370 (7)	0.0375 (7)	0.0038 (5)	0.0035 (5)	−0.0031 (5)
C2	0.0376 (7)	0.0488 (8)	0.0501 (8)	0.0144 (6)	0.0008 (6)	−0.0016 (7)
C3	0.0513 (8)	0.0484 (8)	0.0442 (8)	0.0120 (7)	−0.0065 (6)	0.0047 (6)
C4	0.0572 (9)	0.0476 (8)	0.0345 (7)	0.0076 (7)	0.0028 (6)	0.0034 (6)
C5	0.0457 (8)	0.0419 (7)	0.0356 (7)	0.0095 (6)	0.0075 (6)	−0.0007 (6)
C6	0.0338 (6)	0.0312 (6)	0.0334 (6)	0.0043 (5)	0.0040 (5)	−0.0026 (5)
C7	0.0363 (6)	0.0317 (6)	0.0350 (6)	0.0052 (5)	0.0076 (5)	−0.0005 (5)
C8	0.0563 (9)	0.0386 (7)	0.0445 (8)	0.0131 (6)	0.0188 (7)	0.0076 (6)
C9	0.0404 (8)	0.0763 (12)	0.0702 (12)	0.0155 (8)	0.0231 (8)	0.0002 (10)
N1	0.0331 (6)	0.0560 (7)	0.0470 (7)	0.0112 (5)	0.0137 (5)	0.0033 (6)
N2	0.0440 (6)	0.0422 (6)	0.0437 (6)	0.0151 (5)	0.0122 (5)	0.0063 (5)
N3	0.0554 (8)	0.0581 (9)	0.0595 (9)	0.0307 (7)	0.0210 (7)	0.0176 (7)
O1	0.0558 (7)	0.0577 (7)	0.0402 (6)	−0.0082 (5)	0.0041 (5)	−0.0058 (5)
O2	0.0627 (7)	0.0715 (8)	0.0479 (7)	0.0109 (6)	0.0288 (6)	0.0109 (6)
S1	0.0408 (2)	0.0428 (2)	0.0345 (2)	0.00504 (13)	0.01310 (14)	0.00140 (12)

### Geometric parameters (Å, °)

**Table:**

C1—C2	1.390 (2)	C8—S1	1.7532 (15)
C1—C6	1.4068 (19)	C8—H8A	0.9700
C1—N1	1.4234 (19)	C8—H8B	0.9700
C2—C3	1.380 (2)	C9—N1	1.4616 (19)
C2—H2	0.9300	C9—H9A	0.9600
C3—C4	1.373 (2)	C9—H9B	0.9600
C3—H3	0.9300	C9—H9C	0.9600
C4—C5	1.381 (2)	N1—S1	1.6338 (14)
C4—H4	0.9300	N2—N3	1.3721 (18)
C5—C6	1.398 (2)	N3—H1N	0.86 (2)
C5—H5	0.9300	N3—H2N	0.790 (19)
C6—C7	1.4761 (18)	O1—S1	1.4278 (12)
C7—N2	1.2801 (17)	O2—S1	1.4270 (12)
C7—C8	1.5063 (19)		
			
C2—C1—C6	119.61 (14)	S1—C8—H8A	109.6
C2—C1—N1	119.68 (13)	C7—C8—H8B	109.6
C6—C1—N1	120.71 (13)	S1—C8—H8B	109.6
C3—C2—C1	121.03 (14)	H8A—C8—H8B	108.1
C3—C2—H2	119.5	N1—C9—H9A	109.5
C1—C2—H2	119.5	N1—C9—H9B	109.5
C4—C3—C2	120.07 (14)	H9A—C9—H9B	109.5
C4—C3—H3	120.0	N1—C9—H9C	109.5
C2—C3—H3	120.0	H9A—C9—H9C	109.5
C3—C4—C5	119.60 (15)	H9B—C9—H9C	109.5
C3—C4—H4	120.2	C1—N1—C9	120.59 (14)
C5—C4—H4	120.2	C1—N1—S1	117.21 (10)
C4—C5—C6	121.84 (14)	C9—N1—S1	118.42 (13)
C4—C5—H5	119.1	C7—N2—N3	119.27 (13)
C6—C5—H5	119.1	N2—N3—H1N	118.0 (13)
C5—C6—C1	117.81 (13)	N2—N3—H2N	108.4 (14)
C5—C6—C7	120.23 (12)	H1N—N3—H2N	121 (2)
C1—C6—C7	121.95 (13)	O2—S1—O1	117.61 (8)
N2—C7—C6	118.14 (12)	O2—S1—N1	107.94 (8)
N2—C7—C8	122.12 (12)	O1—S1—N1	111.81 (8)
C6—C7—C8	119.74 (11)	O2—S1—C8	111.06 (8)
C7—C8—S1	110.19 (10)	O1—S1—C8	107.46 (8)
C7—C8—H8A	109.6	N1—S1—C8	99.48 (8)
			
C6—C1—C2—C3	−0.9 (2)	C6—C7—C8—S1	−33.65 (17)
N1—C1—C2—C3	179.38 (15)	C2—C1—N1—C9	9.0 (2)
C1—C2—C3—C4	−0.9 (3)	C6—C1—N1—C9	−170.69 (15)
C2—C3—C4—C5	1.7 (3)	C2—C1—N1—S1	−148.76 (13)
C3—C4—C5—C6	−0.7 (3)	C6—C1—N1—S1	31.55 (18)
C4—C5—C6—C1	−1.1 (2)	C6—C7—N2—N3	178.54 (14)
C4—C5—C6—C7	178.92 (14)	C8—C7—N2—N3	−0.9 (2)
C2—C1—C6—C5	1.9 (2)	C1—N1—S1—O2	−172.78 (11)
N1—C1—C6—C5	−178.42 (13)	C9—N1—S1—O2	28.97 (17)
C2—C1—C6—C7	−178.15 (13)	C1—N1—S1—O1	56.36 (14)
N1—C1—C6—C7	1.5 (2)	C9—N1—S1—O1	−101.89 (15)
C5—C6—C7—N2	2.4 (2)	C1—N1—S1—C8	−56.87 (13)
C1—C6—C7—N2	−177.54 (13)	C9—N1—S1—C8	144.88 (15)
C5—C6—C7—C8	−178.13 (13)	C7—C8—S1—O2	169.33 (11)
C1—C6—C7—C8	1.9 (2)	C7—C8—S1—O1	−60.75 (13)
N2—C7—C8—S1	145.78 (13)	C7—C8—S1—N1	55.82 (12)

### Hydrogen-bond geometry (Å, °)

**Table:**

*D*—H···*A*	*D*—H	H···*A*	*D*···*A*	*D*—H···*A*
N3—H1N···O1^i^	0.86 (2)	2.46 (2)	3.221 (2)	147.7 (17)
N3—H2N···N2^ii^	0.790 (19)	2.376 (19)	3.094 (2)	151.8 (19)
C8—H8A···O1^i^	0.97	2.59	3.4178 (19)	144.

Symmetry codes: (i) −*x*+5/2, *y*−1/2, −*z*+1/2; (ii) −*x*+3, −*y*, −*z*.
